# Rhizosphere Microbiome: The Emerging Barrier in Plant-Pathogen Interactions

**DOI:** 10.3389/fmicb.2021.772420

**Published:** 2021-10-29

**Authors:** Jingtao Li, Chenyang Wang, Wenxing Liang, Sihui Liu

**Affiliations:** ^1^Key Lab of Integrated Crop Pest Management of Shandong Province, College of Plant Health and Medicine, Qingdao Agricultural University, Qingdao, China; ^2^College of Science and Information, Qingdao Agricultural University, Qingdao, China

**Keywords:** rhizosphere microbiome, recruitment, pathogens, plant immunity, root exudates

## Abstract

In the ecosystem, microbiome widely exists in soil, animals, and plants. With the rapid development of computational biology, sequencing technology and omics analysis, the important role of soil beneficial microbial community is being revealed. In this review, we mainly summarized the roles of rhizosphere microbiome, revealing its complex and pervasive nature contributing to the largely invisible interaction with plants. The manipulated beneficial microorganisms function as an indirect layer of the plant immune system by acting as a barrier to pathogen invasion or inducing plant systemic resistance. Specifically, plant could change and recruit beneficial microbial communities through root-type-specific metabolic properties, and positively shape their rhizosphere microorganisms in response to pathogen invasion. Meanwhile, plants and beneficial microbes exhibit the abilities to avoid excessive immune responses for their reciprocal symbiosis. Substantial lines of evidence show pathogens might utilize secreting proteins/effectors to overcome the emerging peripheral barrier for their advantage in turn. Overall, beneficial microbial communities in rhizosphere are involved in plant–pathogen interactions, and its power and potential are being explored and explained with the aim to effectively increase plant growth and productivity.

## Introduction

In the engagement with plants, phytopathogens have evolved sophisticated invasion strategies, for their own benefits, to bypass defense system and efficiently infect the hosts. As a counterpart, in order to stay healthy, plants have developed powerful weapons to ward off pathogens, including the well-studied multilayered physical barriers, preformed defenses, and innate immune system (Zhang et al., 2020). Recent accumulating studies demonstrate that some pathogens could be blocked by another line of surveillance system, an emerging defense barrier, the plant microbiome, which could be separated as the phyllosphere microbiome and the rhizosphere microbiome (Hacquard et al., 2017; Gong and Xin, 2021). Rhizosphere microbiome, known as the second genome of plants, collectively containing bacteria, fungi, and oomycetes, are closely related to plant growth and health (Berendsen et al., 2012; Mueller and Sachs, 2015; Cai et al., 2017; Wu et al., 2018). The typical functional groups, such as rhizobia, mycorrhizal fungi, and the pathogenic microbes, of rhizosphere microorganisms, affecting plant growth and health, have been well studied in the past few decades (van der Putten et al., 2007; Raaijmakers et al., 2009; Dardanelli et al., 2011; Mendes et al., 2013; Tedersoo et al., 2020), while the interaction between plants and other rhizosphere microbial communities is less well-understood (Berendsen et al., 2012; Tedersoo et al., 2020). These plant microbial groups show potential functions related to probiotics and plant protection, attracting attention from research community; however, how the rhizosphere microbial communities influence plant growth and resistance remains scarce (Hacquard et al., 2017).

Traditional culture-dependent approaches, the developed next-generation sequencing (NGS) and the meta-omics technology have served as a key tool for profiling microbial assemblages. Studies suggest that plants affect and recruit soil beneficial microbial community in response to pathogenic microorganism attack, without activating a strong immune response to support its growth and fitness (Hacquard et al., 2017; Yin et al., 2021). Moreover, substantial work has revealed that plants could distinguish pathogenic and beneficial microbes accurately and maintain the dynamic balance between plant growth and defenses (Hacquard et al., 2017; Bozsoki et al., 2020; Zhou et al., 2020; Buscaill and van der Hoorn, 2021; Emonet et al., 2021; Ma et al., 2021; Zhang et al., 2021). Here we review and discuss (i) the current status of rhizosphere microbiome; (ii) emphasizing on its role in the context of plant-pathogen interactions, by acting as a barrier to pathogen invasion; (iii) showing the possibility of engineering disease-suppressive microbes in response to pathogen attack.

## Rhizosphere Microbiome and Plant Disease Management

The diverse surrounding environment formed by soil texture favors the coexistence of a wide-range of microorganisms including bacteria, archaea, fungi, oomycetes, viruses, and protists, all of which interact with each other in complex trophic exchange networks (Compant et al., 2019; Wei et al., 2019; Fitzpatrick et al., 2020). Rhizosphere microorganisms can be beneficial or harmful to the host plant health (Yu et al., 2019a). The harmful microbes, such as soil-borne pathogens, reduce plant growth, cause yield loss, and threaten agricultural production which has been widely studied for decades (Yin et al., 2021). However, beneficial microbes (including mutualistic microbes) can promote plant growth by increasing nutrient availability, producing plant hormones, and enhancing tolerance to biotic and abiotic stresses (Haney et al., 2015; Rolli et al., 2015; Jacoby et al., 2017; Yin et al., 2021). We focus on the beneficial microbial events in favor of plant protection against pathogen attack.

**Figure 1:**
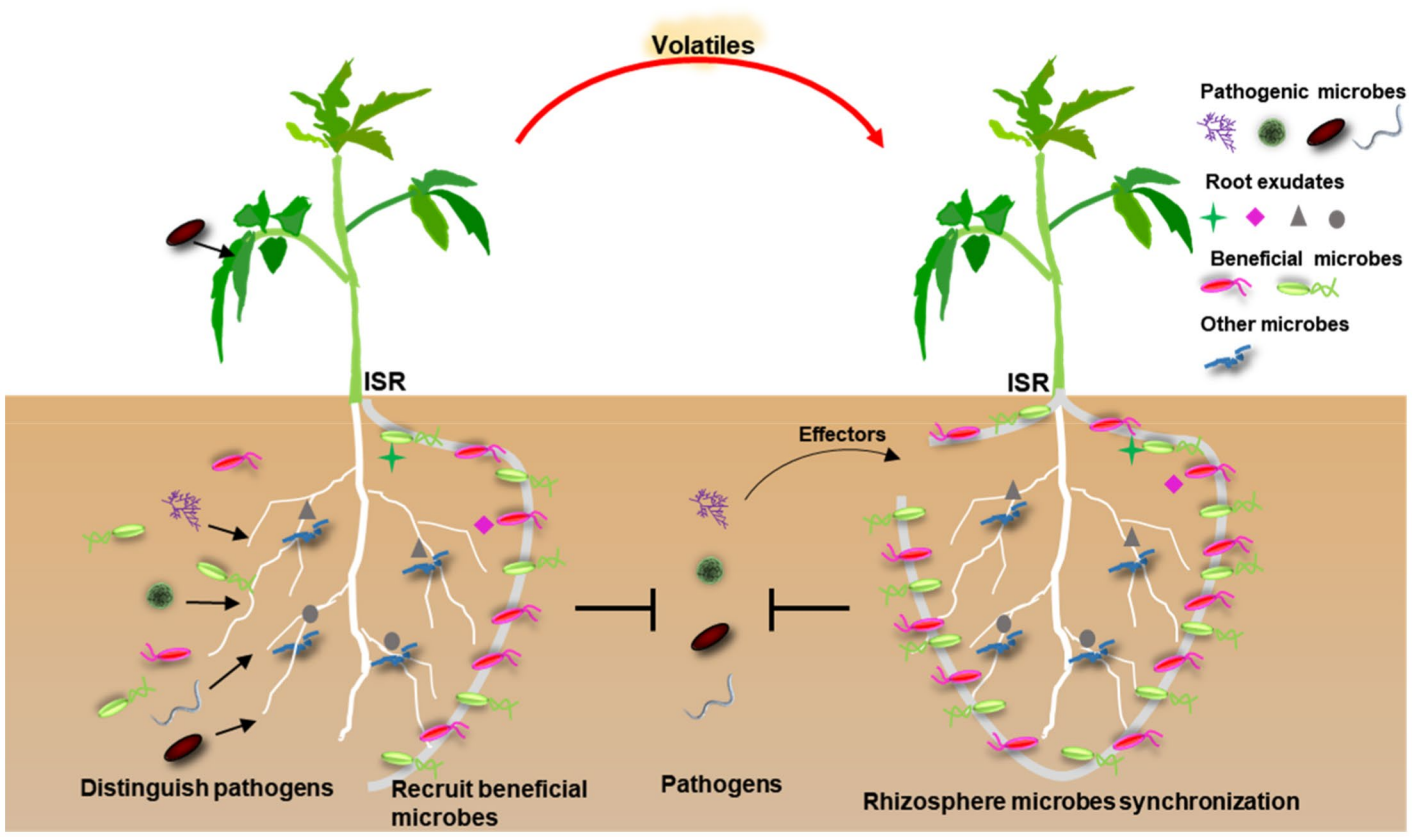
A conceptual model of pathogen-mediated rhizosphere microbial recruitment for plant protection. First, the predecessor plants release root exudates into soil to manipulate soil microbial community dynamics or specifically recruit beneficial microbes after precisely recognizing pathogens invasion. The resulting microbial recruitments could effectively avoid excessive immune responses and then directly inhibit pathogens or induce systemic resistance (ISR). The surrounding plants possibly recruit rhizosphere microbiota through aerial signals (volatiles) from diseased plants. Successful pathogens use effectors, or other strategies, to break down the barrier formed by rhizosphere microbes for their advantage.

Beneficial rhizosphere microbes directly protect plants against pathogens mainly through antagonism, niches and resource competition, or microbial diversity (Berendsen et al., 2012; Hacquard et al., 2017; Kwak et al., 2018; Yin et al., 2021). For the symbiotic fungi, mycorrhizas could benefit plants by providing enhanced nutrient access and tolerance to stress or pathogens (Smith and Read, 2008; Tedersoo et al., 2020). Mycorrhizal fungi also mediate plant interactions with other soil microbes, including pathogens and mycorrhizosphere mutualists that produce vitamins and protect against antagonists (Tedersoo et al., 2020). For instance, ectomycorrhiza (EcM) fungi provide substantial protection against soil-borne pathogens by ensheathing feeder roots and acidifying soil (Tedersoo et al., 2020). In addition, wheat specifically attracts beneficial rhizosphere bacterial microbes [e.g., *Chitinophaga*, *Pseudomonas*, *Chryseobacterium*, and *Flavobacterium*, and a group of plant growth-promoting (PGP) and nitrogen-fixing microbes, including *Pedobacter*, *Variovorax*, and *Rhizobium*], collectively displaying antagonistic activities to the soil-borne pathogens *Rhizoctonia solani* AG8 (Yin et al., 2021). Besides, *Janthinobacterium* displayed broad antagonism against soil-borne pathogens *Pythium ultimum*, *R. solani* AG8, and *R. oryzae in vitro*, and the disease suppressive activity to *R. solani* AG8 in soil (Yin et al., 2021). Furthermore, susceptible cucumber plants against *Fusarium* tend to assemble beneficial microbes (e.g., *Comamonadaceae* and *Xanthomonadaceae*) to control *Fusarium* wilt disease by secreting more organic acids (Wen et al., 2020). Through further growth inhibition assay, *Comamonadaceae*, *Pseudomonas*, and *Stenotrophomonas* are shown to reduce the growth of *F. oxysporum in vitro* (Wen et al., 2020). Though none of flavobacterial isolates directly displayed antibacterial activity toward *R. solanacearum* on solid media, the specific monosaccharide transporters in flavobacteria possibly uptake monosaccharides and reduce the availability of sugars to which *R. solanacearum* lectin binds and thereby reduces infection of tomato under cultured conditions (Kwak et al., 2018). These observations suggest that rhizosphere microbiome selection could accumulate beneficial microbes to directly inhibit pathogens and enhance crop productivity (Figure 1).

In addition to the direct effects on deleterious microbes in the rhizosphere, many beneficial rhizosphere microorganisms have been found to boost the defense capacity of plants. Studies show that plant microbiota can accelerate activation of plant defense in the manner of induced systemic resistance (ISR; Raaijmakers et al., 2009; Dardanelli et al., 2011; Berendsen et al., 2012; Mendes et al., 2013). For example, the plant growth promoting rhizobacterium (PGPR) *Pseudomonas fluorescens* WCS417 could induce ISR in *Arabidopsis* by accelerating defense-related gene expression and increasing callose deposition at the site of pathogen entry (Van der Ent et al., 2009). Similarly, root inoculation with *P. putida* KT2440 induces systemic resistance in maize plants against the maize anthracnose fungus *Colletotrichum graminicola* by triggering the release of plant volatiles and their transmission from one plant to another (Planchamp et al., 2015). Besides, mycorrhizal fungi, *Trichoderma* spp. and other fungal biocontrol agents have also been found to induce ISR in different plant species (Berendsen et al., 2012). In addition to directly inducing systemic resistance, mycorrhizal fungi (e.g., *Glomus mosseae*) can convey a resistance-induced signal to neighboring tomato plants through underground common mycorrhizal networks (Song et al., 2010). Notably, the well-understood plant-beneficial microbes including nitrogen-fixing rhizobia, PGPR, and mycorrhiza can also modify plant volatiles to induce plant defense (Kong et al., 2021). Given their diffusivity, these microbe-induced plant volatiles (MIPVs) may potentially transmit the status of infected plants to adjacent and distant plants, and elicit plant immune responses in surrounding plants.

Overall, these observations suggest that protective rhizosphere microbes have strong effects on plant health upon pathogen invasion by directly combating pathogens and/or enhancing host ISR, which contribute to disease suppressiveness (Figure 1). Therefore, understanding how plants influence beneficial rhizosphere microbial structure, microbe–microbe interactions, and ultimately influencing all aspects of plant protection, is of great agronomic interest.

## Pathogen-Mediated Rhizosphere Microbiota Recruitment

### Soil-Borne Pathogen-Mediated Recruitment

The soil-borne phytopathogens cause severe damages to plant roots resulting in significant agricultural yield loss. Recent studies revealed plants are capable of recruiting specialized associated microbiome as an adaptation strategy to growth promotion and pathogen protection, by potentially antagonizing pathogens or modulating the host immune system (Berendsen et al., 2012; Wen et al., 2021; Yin et al., 2021). Due to agricultural importance, a few studies in crop and microbiome interactions, upon certain pathogen attacks, provide several lines of good examples. For instance, wheat could recruit specific *Pseudomonas* species, producing antimicrobial compounds, in response to “take-all” disease (Weller et al., 2002). Similarly, compared to the health status wheat, the rhizosphere microbial taxa, where wheat root infection by *R. solani*, are rich in the families such as Enterobacteriaceae, Flavobacteriaceae, Caulobacteraceae, Chitinophagaceae, and Pseudomonadaceae (Poudel et al., 2016). In addition, a recent work shows multi-cycle wheat plantings with soil-borne fungal pathogen *R. solani* AG8 can recruit/enrich beneficial or antagonistic microorganisms to suppress pathogens in the rhizosphere (Yin et al., 2021). Another good example is that barley plants control their rhizosphere community by specifically recruiting antifungal microbes when challenged with *Fusarium graminearum* (Dudenhöffer et al., 2016).

### Foliar Pathogen-Mediated Recruitment

In addition to soil-borne pathogens, it has been found plants recruit beneficial rhizosphere communities through releasing specific root exudates upon foliar pathogen invasion, suggesting an indirect recruitment manner (Yuan et al., 2018). For example, *Arabidopsis* specifically promotes three bacterial species (i.e., *Microbacterium*, *Stenotrophomonas*, and *Xanthomonas* sp.) in the rhizosphere upon foliar defense activation by the downy mildew pathogen *Hyaloperonospora arabidopsidis* (Berendsen et al., 2018). Another case study is that *Arabidopsis* could recruit the beneficial bacterium *Bacillus subtilis* upon the foliar pathogen *P. syringae* pv*. tomato* (*Pst*) invasion (Rudrappa et al., 2008). In a recent study, *Arabidopsis* can recruit beneficial rhizosphere community *via* modification of plant exudation patterns (e.g., amino acids, nucleotides, and long-chain organic acids) in response to exposure to *Pst*, to elicit a disease-suppressive response (Yuan et al., 2018). Further study reveals that root-secreted amino acids and long-chain fatty acids stimulated by *Pst* infection can attract soil specific *Pseudomonas* populations, contributing to plant resistance against aboveground pathogen attack through the induction of plant ISR (Wen et al., 2021).

### Pathogen-Mediated Distant Rhizosphere Microbiota Recruitment

Infection of plants by microbial pathogens, such as virus, bacteria, and fungi, elicits the release of MIPVs (Kong et al., 2021), among the volatile organic compounds (VOCs), representing one of the many plant-to-plant signaling systems (Heil and Ton, 2008). For example, activation of Salicylic acid (SA) synthesis and subsequent signaling has been found in healthy plants exposed to volatiles (such as hexenal isomers and 2,3-butanediol) produced by spatially distant apple plants infected with *Erwinia amylovora* (Cellini et al., 2018). As SA is one modulator of the rhizosphere microbiome assembly (Lebeis et al., 2015; Berendsen et al., 2018), it is deductive that pathogen infection would result in differential stimulation of specific microbiota in surrounding healthy plant rhizosphere by VOCs and activated SA. Moreover, leaves of the tomato plant treated with a model PGPR, *Bacillus amyloliquefaciens* GB03, released β-caryophyllene as a signature VOC, which elicited the release of a large amount of SA in the root exudates of a neighboring tomato seedling (Kong et al., 2021). Intriguingly, the rhizosphere microbiota diversity of the PGPR-treated emitter plant was highly similar to that of its neighboring receiver plant (Kong et al., 2021). Therefore, the pathogen infection could potentially shape rhizosphere microbiota of neighboring plants through direct MIPV, or through pathogen-mediated PGPR recruitment in rhizosphere and subsequent MIPV. However, more direct evidence is required to prove these hypotheses.

Over all, these discoveries indicate a tight linkage between the microbial community in rhizosphere and pathogen infection, and provide the possibility that plants actively recruit disease-suppressive microbes in response to pathogens attack, eventually providing a wide opportunity to suppress disease and increase crop production (Figure 1).

## Factors Governing Plant Rhizosphere Microbiome

The rhizosphere microbial community attached to the root surface is different from the microbes in the non-rhizosphere soil, indicating microbial community establishment in the rhizosphere is not random but rather driven by host plant selection (Yin et al., 2021). During plant growth, 5–21% of their photosynthetically fixed carbon are secreted into rhizosphere micro-domain through the roots, serving as important nutrient source of soil microbial community and affecting the assembly process of plant rhizosphere (Li et al., 2019). Currently, it is widely accepted that microbial communities are tightly associated with plant roots (Bulgarelli et al., 2013; Compant et al., 2019). In addition, roots dominate the qualitative and quantitative compositions of root exudates, a complicated form of fluids emitted through the roots, depending on the plant genotype/species, developmental stage, abiotic, and biotic stresses (Lundberg et al., 2012; Chaparro et al., 2014; Bulgarelli et al., 2015; Tkacz et al., 2015; Yin et al., 2021).

Root exudates such as sugars, organic acids (e.g., long-chain fatty acids, short-chain organic acids, amino acids, and plant volatiles), metabolites, phytohormones, and complex mucus-like polymers are crucial in attracting and selecting microorganisms, thus altering the composition and structure of rhizosphere microbes (Broeckling et al., 2008; Carvalhais et al., 2015; Berendsen et al., 2018; Sasse et al., 2018; Yuan et al., 2018; Wen et al., 2020, 2021; Kong et al., 2021). For instance, long-chain fatty acids and amino acids were identified to play important roles in the recruitment of potentially beneficial microbes (e.g., *Pseudomonas* populations; Yuan et al., 2018; Wen et al., 2021). A recent work revealed that four short-chain organic acids (citric acid, pyruvate acid, succinic acid, and fumarate) were released at higher abundance, which may be responsible for the enrichment of *Comamonadaceae*, a potential beneficial microbial group (Wen et al., 2020), while root-secreted malic can recruit beneficial *Bacillus* to the rhizosphere (Rudrappa et al., 2008). The resultant ratio and composition of both sugars and phenolics in the root exudates have a profound effect on natural soil microbial composition (Badri et al., 2009), and the addition of a phenolic acid, *p*-coumaric acid, to the soil influences soil microbial communities of cucumber rhizosphere (Zhou and Wu, 2012). Besides, phenolics could increase abundance of bacteria or PGPR in *Arabidopsis* rhizosphere (Badri et al., 2009). In addition, *Arabidopsis* produces a range of specialized triterpenes that direct the assembly of specific root microbiota, enabling to shape and tailor the microbial community around its roots (Huang et al., 2019). Benzoxazinoids released by maize through roots drive plant performance and defense by shaping rhizosphere microbiota (Hu et al., 2018). Flavonoids have been considered crucial root-rhizosphere signal molecules modulating interaction of roots with microorganisms (e.g., rhizobia, mycorrhizal fungi, root pathogens or pests ranging from bacteria to fungi and insects, and nematode; Hassan and Mathesius, 2012). For instance, root-derived flavones enrich rhizosphere *Oxalobacteraceae* taxa to improve maize growth and nitrogen acquisition, implying that flavonoid-mediated root-microbe interactions might also modulate developmental processes in the host plants (Yu et al., 2021). Furthermore, a recent work reveals that receptor kinase *FERONIA*-mediated ROS production regulates levels of beneficial *Pseudomonas* in the rhizosphere microbiome (Song et al., 2021). The defense-related phytohormones SA and jasmonic acid (JA) are important in modulating the rhizosphere microbial assembly of *Arabidopsis*, and deletion of JA or SA biosynthesis genes altered the rhizosphere microbial community of plants (Carvalhais et al., 2015; Lebeis et al., 2015). Because biotrophic or necrotrophic pathogens systemically accumulate SA or JA, respectively (Berendsen et al., 2018), thereby pathogen infection is suggested to affect rhizosphere microbiome assembly by phytohormones. To further support it, the infected plants by *Pst* exhibited significantly higher exudation of amino acids, nucleotides, and long-chain organic acids, which play roles in the establishment of beneficial rhizosphere communities (Yuan et al., 2018). Therefore, root exudates serve as important chemical tools to manipulate the rhizosphere microbial community, depending on specific host and environmental conditions including pathogen invasion (Sasse et al., 2018; Kong et al., 2021). However, the effects of root exudates on rhizosphere microbial communities are highly variable, complex, and dynamic (Yin et al., 2021). Our understanding of how plants shape rhizosphere microorganism assembly by root exudates, especially the plant-derived molecules following pathogen attack, is not fully clear.

## Balance Between Plant Immunity and Distinction of Pathogens From the Rhizosphere Microbiome

Plants have a genetically imprinted innate immune system, to prepare for the challenging of pathogenic and beneficial organisms, that responds to microbe-associated molecular patterns (MAMP), as perceived by the host cell surface-localized pattern recognition receptors (PRRs; Zipfel, 2008). Efficient immune responses can help plants to achieve self-protection and contribute to the maintenance of a stable microorganism community (Yin et al., 2021), while excessive immune responses lead to the inhibition of plant growth and affect the colonization of other beneficial microorganisms (Ma et al., 2021). Avoiding MAMP-triggered excessive plant immunity and accurately distinguishing pathogens is crucial.

**Figure 2:**
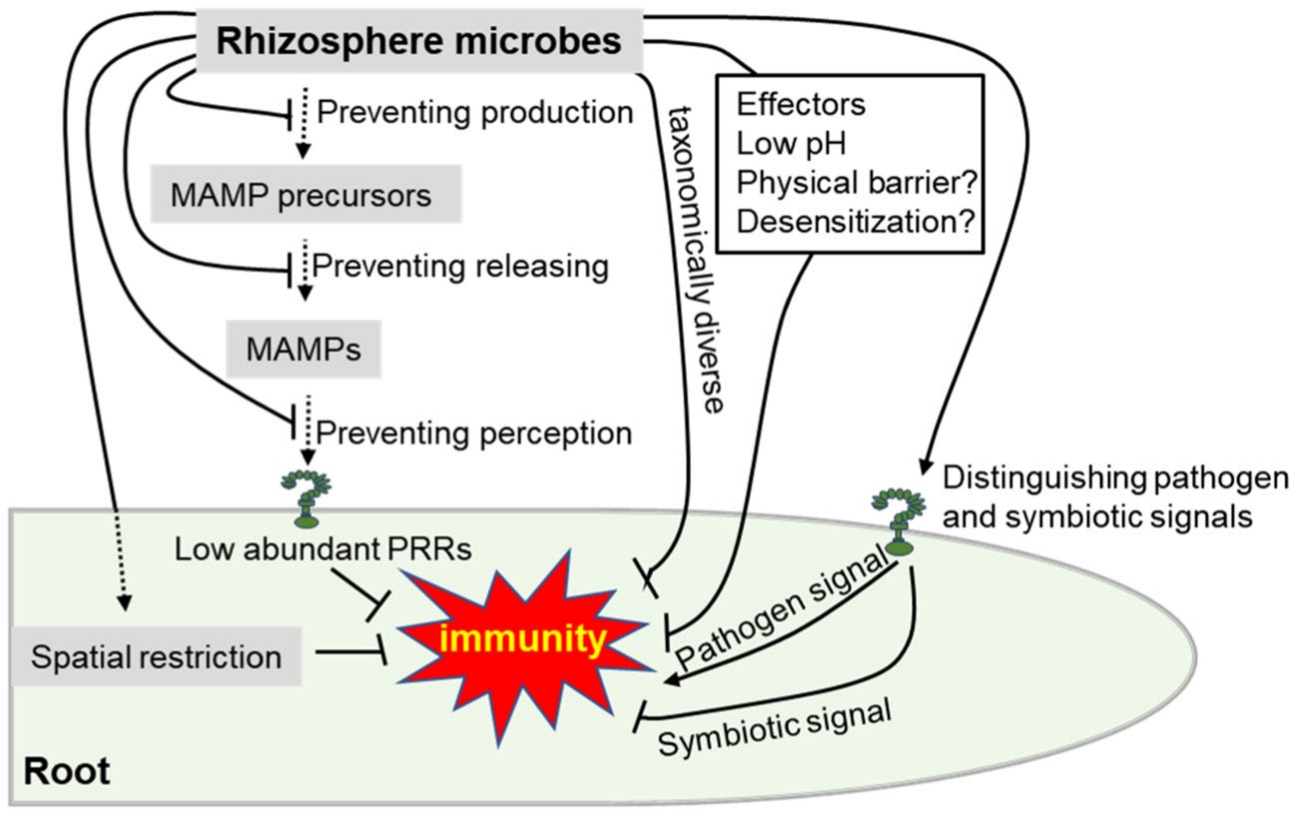
Schematic overview of plant and rhizosphere microbes that evade or suppress root immune responses as described in the main text. Briefly, beneficial microbes can suppress plant immune response by evading pattern recognition receptor (PRR) recognition of MAMPs, taxonomically diverse, secreted effectors, low pH, physical barrier, desensitization and symbiosis-related molecules. On the other hand, plants could also suppress root immune responses by immune spatial restriction, low abundant PRRs and precisely distinguishing between the pathogen and beneficial microbes. Beneficial rhizosphere microbes can evade or suppress root immunity, suggesting that this is a useful trait for rhizosphere inhabitants.

To colonize plants, adapted beneficial microorganisms including pathogens have adopted nine extracellular strategies to avoid recognition by PRRs, which occur at three levels (Figure 2): preventing MAMP production (i.e., polymorphisms in protein MAMPs, polymorphisms in glycan MAMPs, and downregulating MAMP production), preventing MAMP release (i.e., hiding MAMP precursors with proteins, shielding MAMP precursors with glycans, blocking MAMP release by inhibiting the activity of host hydrolases, and disintegrating host-derived hydrolases), and preventing MAMP perception (i.e., degrading MAMPs and sequestering released MAMPs; Buscaill and van der Hoorn, 2021). In fact, root immune responses are generally lower than in the shoot, in part because of low abundance or absence of PRRs (Beck et al., 2014; Emonet et al., 2021). Moreover, spatial restriction of meristematic activity and immune responses are thought to be necessary to adequately balance growth and constitutive immune responses to rhizosphere microbiota; a recent study divided the root meristem into a central zone refractory to FLS2 expression and a cortex that is sensitized by FLS2 expression, causing flagellin-dependent collapse and growth inhibition upon commensal bacteria stimulation (Emonet et al., 2021). Furthermore, plants restrict their defense to vulnerable regions with broken endodermal barriers or absent, such as the elongation zone or lateral root emergence sites, where bacteria are found to preferentially accumulate (Faulkner and Robatzek, 2012; Zhou et al., 2020). In addition, taxonomically diverse root bacterial commensals suppress the inhibition of *Arabidopsis* root growth triggered by MAMPs without affecting the effective resistance to the pathogenic microorganisms (Ma et al., 2021). Recent evidence suggests that plant-beneficial *Pseudomonas* spp. suppresses flg22-induced root immunity by producing gluconic acid that lowers the environmental pH (Yu et al., 2019b). More than that physical barriers provided by mucus and induced desensitization of epithelial cells to bacterial lipopolysaccharide are present to avoid aberrant activation of the animal immune system (Lathrop et al., 2011; Berendsen et al., 2012; Chinen and Rudensky, 2012), which might be similarly exploited for rhizosphere microbiome. In addition to pathogens, beneficial microbes can also interfere with different host immune signaling components by secreting proteins/effectors (Yu et al., 2019a). However, both symbiotic microorganisms and pathogenic microorganisms could still equally trigger plant immune responses by MAMPs (Zhou et al., 2020). Hence, the ability to precisely recognize non-self-patterns, to respond to pathogens or beneficial microbes, and to maintain the dynamic balance between beneficial association and plant defense is still essential for plants (Yin et al., 2021).

In plants, innate immune systems can prevent most pathogens, while allowing colonization of symbiosis and beneficial microbes (Bozsoki et al., 2020; Zhou et al., 2020; Fröschel et al., 2021; Zhang et al., 2021). For example, after inoculation with the vascular pathogen *Verticillium longisporum*, pathogenic oomycete *Phytophthora parasitica*, or mutualistic endophyte *Serendipita indica*, plant root cell-layer responses were different, as revealed by cell-layer translatomes analysis, reflecting the fundamentally different colonization strategies of these microbes (Fröschel et al., 2021). In addition, it was recently shown that the root has an inherently dampened MAMP response until it encounters damage, which locally boosts immune responsiveness (Zhou et al., 2020). In other words, the expression of immune receptors in the healthy root cells of *Arabidopsis* was extremely low when interacting with beneficial or harmless microorganisms. When the root cells are damaged by pathogen invasion, the adjacent root cells begin to express the immune receptors and respond to MAMPs quickly, activating the immune responses precisely near the infection point (Zhou et al., 2020). These findings, for the first time, reveal how plants control immune receptors, and it is of great significance for future research to integrate two different signals, damage and MAMPs, to distinguish different microorganisms. Besides, a recent study shows that plants have evolved lysine motif (LysM) receptors (CERK6, NFR1) to recognize chitin and nodulation (Nod) factors. Though the protein structures of CERK6 and NFR1 are very similar, plants use regions II and IV of LysM1 to specifically recognize pathogens (chitin) or symbiotic signaling molecules (Nod factor) and initiate differential signaling of immunity or root nodule symbiosis (Bozsoki et al., 2020). Another recent advance revealed that the CO4 (Chitotetraose) symbiotic receptor OsMYR1 can initiate symbiotic signaling as well as repress rice immunity by depleting the receptor-like kinase OsCERK1, thereby preventing the formation of the immunity complex OsCERK1-OsCEBiP in rice (Zhang et al., 2021), suggesting that OsMYR1 and OsCEBiP receptors compete to bind OsCERK1 to determine the specific response outcomes of symbiosis and immunity signals. Therefore, these lines of evidence suggest plants encountering various microbes in nature could respond appropriately to pathogenic or symbiotic microbes (Figure 2), and the exploration of plant distinguishing pathogen from rhizosphere microbiome is likely to be revealed.

## Prospects of Rhizosphere Microbiome As a Barrier

The accumulating lines of evidence suggest that microbial networks, formed on healthy plant tissues, function as an indirect layer of the plant immune system by acting as a barrier to pathogen invasion, which might help explain why plant disease remains an exception in the natural environment (Hacquard et al., 2017). To compete, many microorganisms in the rhizosphere produce antimicrobial compounds targeting specific microbes including pathogens, a process known as biocontrol activity (Whipps, 2001). The idea that a healthy microbiome can protect plants from pathogen infection and can biologically control diseases, has been well documented in the case of disease-suppressive soils (Berendsen et al., 2012; Hacquard et al., 2017). For example, exploiting the rhizosphere microbiota in plant resistance against fungal pathogens has been reported; the inoculation of *Nicotiana attenuata* seeds with a root-associated bacteria community efficiently protects the plant against the sudden-wilt fungal disease under both laboratory and field conditions (Santhanam et al., 2015). Similarly, comparative analyses of rhizosphere metagenomes from resistant and susceptible tomato plants enabled the identification of more abundant *Flavobacterium* in the resistant plant rhizosphere microbiome and, as a proof of concept, the identified bacterial strains could suppress *Ralstonia solanacearum* bacterial wilting disease development in a susceptible plant in pot experiments (Kwak et al., 2018). Therefore, artificial enrichment of beneficial taxa in the laboratory or in the field can promote growth and protect plants from biotic stresses (Lugtenberg and Kamilova, 2009; Berendsen et al., 2012; Song et al., 2021).

On the other hand, increasing strain richness, for example within the biocontrol species *P. fluorescens*, can also cause community collapse and the subsequent loss of plant protection (Becker et al., 2012). In addition, some plant pathogens evolved mechanisms to counteract the beneficial recruitment, resulting in successful infection, through secreting effector proteins (Rovenich et al., 2014; Kettles et al., 2018; Snelders et al., 2018, 2020). For instance, the wheat pathogen *Zymoseptoria tritici* could secrete Zt6 effector, executing important functions in antimicrobial competition and niche protection, potentially due to toxicity (Kettles et al., 2018). The virulence effector VdAve1 from plant fungal pathogen *Verticillium dahliae* displays antimicrobial activity and facilitates the fungal colonization on cotton and tomato through the manipulation of their microbiome by suppressing antagonistic bacteria (Snelders et al., 2020). Usually, pathogens encode large amounts of secreted protein; however, the functions of many effectors in terms of host plant manipulation remain unknown. This might suggest the possible utilization of effectors as exquisite tools for the interaction with other microbes, potentially modulating microbiome compositions (Snelders et al., 2018). Therefore, further understanding of the functional interaction of pathogens, plants, and their microbial chaperones remains urgently required, with the aim to effectively promote plant growth and productivity.

## Concluding Remarks

High-throughput rhizosphere microbiome profiling, combined with perturbation experiments, has shed light on the ecological importance of recruiting specific rhizosphere microorganism for plants against pathogen invasion. These advances have expanded our understanding of plant–microbe interactions, and further research on this topic will contribute significantly one important consideration, utilizing rhizosphere microbiome in disease resistance. However, several pressing questions remain to be addressed. For example, do plants recruit different microbes in response to different pathogens? How plants recruit beneficial microbes through root exudates following sensing pathogens? What root exudates that affect rhizosphere microorganism are directly related to pathogen infection? Do other pathogens contain effector proteins that target rhizosphere microorganism beyond plants? How plants distinguish the commensal microbes and pathogenic microbes through more PRRs or signal pathway? In addition, how could we get further insight into the triangular relationship of plants, beneficial microorganisms, and pathogens?

Recently, one new experimental technique, the holo-omics strategy, that pairs host and microbial datasets was proposed (Nyholm et al., 2020). The experimental designs pair host-centered omic strategies, such as transcriptomics, metabolomics, epigenomics, and proteomics, with the more commonly used microbial-focused techniques, such as amplicon sequencing, shotgun meta-genomic, meta transcriptomics, and exometabolomics (Xu et al., 2021). Such holo-omic studies have the power to resolve the functionality of a plant microbiome ecosystem and provide significant information about microbial approach to improving host health and fitness, which will only increase in the near future.

## Author Contributions

JL and CW wrote the original paper. SL initiated the review and wrote partly for this manuscript. JL and WL provided funding. All authors contributed to the article and approved the submitted version.

## Funding

This work was financially supported by the National Natural Science Foundation of China (31901830 and 31972213), the Natural Science Foundation of Shandong Province (ZR2019BC032 and ZR2020KC003), Shandong Province “Double-Hundred Talent Plan” (WST2018008), and Taishan Scholar Construction Foundation of Shandong Province (tshw20130963).

## Conflict of Interest

The authors declare that the research was conducted in the absence of any commercial or financial relationships that could be construed as a potential conflict of interest.

## Publisher’s Note

All claims expressed in this article are solely those of the authors and do not necessarily represent those of their affiliated organizations, or those of the publisher, the editors and the reviewers. Any product that may be evaluated in this article, or claim that may be made by its manufacturer, is not guaranteed or endorsed by the publisher.
